# Supplementary material for: Learning curve and surgical outcome of robotic assisted colorectal surgery with ERAS program
Source: Sci Rep. 2022 Nov 29;12:20566. doi: 10.1038/s41598-022-24665-w (PMC9709162; doi:10.1038/s41598-022-24665-w)
Supplement: Supplementary file 1 — Supplementary Information 1. [file 41598_2022_24665_MOESM1_ESM.zip › LSC Raw data-Ñ[▒K/054/HS_ñw╝╨░O▒Kñσ.pdf]

正式摘要(電子簽章：000000179956582)

W83 / W83 ☐英文標題(注意:無英文姓名-請補病人基本檔!) ☐【讀取】憑證

臺中榮民總醫院

出院病歷摘要

轉入號： 轉出號：

[redacted]

**(8)**流水編號	
**(9)**入院日期	108/11/01 (2019) 直腸外科 W83 - 053
**(10)**轉科日期	
**(11)**出院日期	108/11/12 (2019) 住院天數計 11 日

診斷

- 入院 : 1) Hypertension under control
  2) AMI S/P PCI with 2x DES on 2018/07
  3) DM uncontrolled
  4)CKD
  5)adenocarcinoma of sigmoid colon
- 出院 : 1) Hypertension under control
  2) AMI S/P PCI with 2x DES on 2018/07 with post op NSTEMI s/p PCI 2019/11/3
  3) DM uncontrolled
  4)CKD
  5)adenocarcinoma of sigmoid colon s/p laparoscopic anterior resection with end to end anastomosis, laparoscopic cholecytstectomy by GS doctor on 2019/11/05

主訴

- intermittent bloody stool for years

病史

- This 59 y/o male has the underlying disease of
  1) Hypertension under control
  2) AMI S/P PCI with 2x DES on 2018/07
  3) DM uncontrolled
  4)CKD

  He suffered from intermittent bloody stool for years and never seek for medical treatment till 2019/07 due to massive bleeding from anus and was informed of low Hemoglobin from regular check up in CV OPD . He came to our CRS OPD for help . He had no epigastralgia, nausea, and decreased appetite. There was no vomiting, change of bowel habit, or body weight loss. He denied fever, URI symptoms, palpitation, or heartburn sensation. Sigmoidscopy revealed Sigmoidscopy was up to 20cm FAV. Colon tumor was noted at 20cm FAV, S/p biopsy & tatto. After discussion with patient, he was admitted for surgical intervension.

  Under the impression of sigmoid colon tumor, he was arranged for Laparoscopic Anterior resection and anastomosis on 2019/11/ 05

體檢發現

- Evaluation

  PE:

  Tumor marker CEA CA-199
  2019/10 4.2
  image sono,
  2019/10 - GBS

  colonsocopy

- 2019

  SE
  2018

  Dx:

  - General appearance :
    - easy looking , conscious clear , without cardiopulmonary distress
    - Vital signs自動帶入date 體溫(BT) 脈搏(P) 呼吸(R) 血壓(BP) 身高(BH) 體重(BW) BMI
      20191101 36.1℃ 75次/分 18次/分 187/82mmHg 164.8cm 83.5kg 30.7
    - Vital signs: B.P. 187/82 mmHg , P.R. 75 /min , R.R. 18 /min , B.T. 36.1 ℃
  - HEENT :
    - Eyes: no anemic conjunctiva, no icteric sclera, normal EOM
    - ENT: no otorrhea, no rhinorrhea, no erythematous mucosa, no injected tonsil, no deformity
  - Neck: supple, freely movable, trachea midline,no deformity, no engorged jugular vein , no palpable thyroid or lymph node enlargement
  - Chest:
    - Inspection: Grossly normal, symmetric and fully expansion, no accessory muscle used, no operation scar, no spider angiomata, no gynecomastia
    - Palpation: no palpable mass or nodule, no subcutaneous emphysema, no tenderness
    - Ausculation: clear breathing sound
  - Heart:
    - Palpation: no thrill, no heave
    - Percussion: normal heart size
    - Ausculation: regular heart beat, normal S1, S2, no gallop, no murmur
  - Extremities: No deformity, full ranged of motion without limitation, no clubbing finger or nail cyanosis, no pitting edema, no ulcer or pigmentation, no obvious skin lesion, no varicose vein, no bone, joint or muscle tenderness

CRS CONDITION

- Karnofsky performance status: (100) Normal, no complaints, no evidence of disease
- PE of Abdomen:
- __1. Inspection: -
- __2. Ausculation: -
- __3. Palapation: -
- __4. Percussion: -
- Digital Rectal Examination: -
- CEA: 4.2 ng/ml
- Imaging Studies:
- Colonoscope report: Sigmoidscopy was up to 20cm FAV. Colon tumor was noted at 20cm FAV, S/p biopsy & tatto.
- Figures:
  1. colon & rectum
  2. colon & rectum
  3. colon & rectum
  4. colon & rectum
  5. colon & rectum

手術日期及方法

- 手術日期及方法20191105 Laparoscopic Cholecystectomy
  20191105 1.Laparoscopic anterior resection with end-to-end anastomosis, double stapling, EndoGIA 60/4.1mm, CDH 29mm, bilateral doughnuts: intact, air leak test: done, no air leak.
  2. High ligation (+) above the left colic artery.
  3. Cut margin: proximal: 12 cm proximal to 1st tumor, distal: 6 cm distal to 3rd tumor
  4. Take down of splenic flexure: yes
  5. Drain: a 12mm penrose drain in pelvis
  6. Repair of mesentery defect:nil

住院治療經過

- After admission, Pre-operation survey was done and there is no contraindication for operation.Patient underwent laparoscopic anterior resection with end to end anastomosis on 2019/11/05 and laparoscopic cholecystectomy on 2019/11/05,In POR, patient suffer chest pain and CXR revealed pulmonary edema and EKG showed LBBB with progressed L5, L6 ST depression S/P PCI without ISR or acute occlusion on 2019/11/6. lab data revealed WBC:8210 RBC:3.56 HGB:10.3 NA:143 K:4.9 BUN:43 CREAT:4.89 GFR:13.02. Due to stable condition, bipap was removed and patient is allowed to discharge with regular OPD follow up ..

合併症

一般檢查

- 累積報告：

DATE	TIME	ALB	TP	BIL.T	BIL.D	ALKP	AST	ALT	LDH	CHOL	TG	HDL-C	LDL	UA	NH3	AMY
1081023	1737						14	15								
1081028	1324	4.4		0.21	0.05	78	14	12	187							
1081101	1720	4.1														
1081104	1514	4.2		0.3				9								
1081105	1928	3.8		0.20	0.07		22	22								
1081109	0737	3.6	6.0	0.17	0.08	51	16	3	239							
1081111	0951	3.3		0.12				3								

DATE	TIME	WBC	RBC	HGB	HCT	MCV	PLT	BLST	NEUT	BAND	SEG	LYM	MON	EOS	BAS
1081023	1737	5340	3.19	9.1	27.8	87.1	186		59.6			28.7	7.7	3.4	0.6
1081028	1324	5380	3.67	10.6	33.0	89.9	192		57.1			31.8	7.4	2.6	1.1
1081104	1541	4320	3.51	10.1			190		56.3			33.8	6.9	2.1	0.9
1081105	1928	8210	3.56	10.3	30.2		191		85.4			10.6	3.4	0.2	0.4
1081107	1218			9.8	30.5										
1081109	0735	5360	3.17	9.2	27.6	87.1	145		67.9			22.0	6.7	3.2	0.2
1081111	0957	3750	2.91	8.5	25.0	85.9	165		59.7			28.3	7.7	3.5	0.8

DATE	TIME	NA	K	CL	CA	BUN	CREAT	Mg	P	CRP	ESR	PCT	LIPASE	LACTATE
1081023	1737	142	5.2	108	7.9	52	5.52							
1081028	1324	138	5.4		7.9	54	5.06							
1081101	1720	140	5.5	113	7.6	55	5.40							
1081104	1514	137	5.5		7.8	50	5.20							
1081105	1928	143	4.9			43	4.89							
1081107	1202	143	4.4		6.8	42	5.70							
1081109	0737	143	4.5	110	7.7	41	5.42							
1081111	0951	141	4.5	111	7.7	41	5.10							

DATE	TIME	GLUCOSE	KETONE	CK	CKMB	PT-P	PT-C	APTT-P	APTT-C	TRO-T
1081023	1737					9.8	10.1	26.3		
1081028	1324	94				9.8	10.1	27.3		
1081105	1928			121	10					58.97
1081105	2246			329	17					377.40
1081106	0335			474	26					1744.00
1081106	0731									2872.00
1081106	0732			508	25					
1081109	2016					10.8	10.1	28.5		

DATE	TIME	PH	PCO2	PO2	SO2	BEB	HCO3	TCO2	HGB	FIO2
1081105	2246	7.311	43.5	116.9	98.0	-4.6	21.5	22.8		

特殊檢查

病理報告

- pending

其它

出院時情況

- 3.改門診治療

出院指示

藥名	劑量	劑量單位	用法	頻次	數量	數量單位	現況	天數	單位
Ulstop tab 20mg	1	TAB	PO	BID	14	TAB	TH	7	天

Ultracet tab	1	TAB	PO	Q8HPRN	9	TAB	TH	3	天
Through tab	1	TAB	PO	QN	7	TAB	TH	7	天
Isobide tab 10mg	1	TAB	PO	TIDAC	21	TAB	TH	7	天

出院計劃

主治醫師 2222H 林俊余 / 住院醫師 9662A 劉冠妤

正式摘要**(電子簽章：000000179956582)**

W83 / W83

doctorRank:B
